# An Unusual, Delayed Presentation of a Migrated Intrauterine Contraceptive Device Into the Rectosigmoid Colon

**DOI:** 10.7759/cureus.42851

**Published:** 2023-08-02

**Authors:** Wuen Lynn Toh, Whui Whui Lim, Wei Keat Andy Tan, Shau Khng Jason Lim

**Affiliations:** 1 Department of Obstetrics and Gynaecology, Singapore General Hospital/ SingHealth, Singapore, SGP

**Keywords:** sigmoidoscopy, contraception, colorectal surgeon, migrated iucd, gynaecology and obstetrics, rectosigmoid colon, intrauterine devices

## Abstract

The current literature suggests that serious complications after intrauterine contraceptive device (IUCD) insertion are rare. We present a rare case of a migrated IUCD into the rectosigmoid colon.

A 33-year-old woman (parity one) presented to the emergency department with a three-day history of flank pain, upper urinary tract infection symptoms, and a low-grade fever. Differentials initially included renal colic or pyelonephritis. However, subsequent computed tomography of the kidneys, ureters, and bladder (CT-KUB) and magnetic resonance imaging of the pelvis (MRI-pelvis) showed a migrated IUCD posterior to the uterine body, with both ends closely abutting onto the adjacent proximal rectum. During further history-taking, she reported a past surgical history of an emergency caesarean section five years ago and the insertion of a copper-IUCD six weeks postnatally. She was subsequently referred to the gynaecologists. In view of the involvement of the bowels, the colorectal surgeons were consulted, and the patient was managed by a multidisciplinary team. The patient subsequently underwent diagnostic hysteroscopy, flexible sigmoidoscopy, diagnostic laparoscopy, removal of impacted IUCD, and repair of the rectum. Intraoperatively, her hysteroscopy noted a normal uterus with an intact cavity. Flexible sigmoidoscopy noted the horizontal arm of the IUCD abutting into the lumen of the rectosigmoid region; however, attempted removal with traction was unsuccessful. A partial rectotomy was done eventually to remove the IUCD.

Migration of an IUCD is rare, with uterine perforation rates ranging from 0.04% to 0.2%. Albeit a rare complication, this case highlights the need for clinicians to be cognizant of complications arising from IUCD insertion, as symptoms are often non-specific and mild. This case also highlights the importance of a multidisciplinary discussion in the management of a migrated IUCD, which may include gynaecologists, colorectal surgeons, radiologists, and more. Many innovative ways were also discussed regarding the assessment of it, which includes preoperative imaging or endoscopic evaluation. Novel methods of removal of migrated IUCD in the rectosigmoid colon have also been proposed, including manual traction, proctoscopy, rigid sigmoidoscopy, and removal via a snare. They provide an alternative to the traditional diagnostic laparoscopy or laparotomy, thus reducing the need for general anaesthesia or operative intervention. Looking forward, long-term studies can be done to evaluate the need for intervention for asymptomatic patients where the risk of surgery may outweigh the benefits.

## Introduction

According to the Centers for Disease Control and Prevention (CDC), family planning is one of the 10 great public health achievements of the 20th century. In 2019, among women of reproductive age, 922 million people used some form of contraception-either traditional or modern methods of contraception. It is estimated that 159 million of them use the intrauterine contraceptive device (IUCD) [1]. With increasing population and education awareness regarding family planning, clinicians should be aware of the complications associated with it.

Migration of an IUCD is rare, with uterine perforation rates ranging from 0.04% to 0.2% [2, 3]. Most perforations occur at the time of IUCD insertion and are asymptomatic [3]. Migration of the IUCD out of the uterus can be associated with serious complications such as haemorrhage and migration to adjacent organs such as the bladder, appendix, bowel, omentum, peritoneal cavity, and retroperitoneum. Literature suggests the most common site of visceral involvement is the omentum, followed by the bladder. Migration into the rectosigmoid is less common [4]. In a review of 356 cases of translocated IUCDs over 15 years, 20 cases of intestinal perforation were reported, of which nine involved the rectosigmoid [5]. There are a few other case reports of translocation of the IUCD into the sigmoid colon as well [4, 6-7]. A retrospective study looking at 78 cases of migrated IUCD in a Finnish hospital noted the median time from insertion to diagnosis to be five months. Forty-nine percent of patients experienced immediate symptoms (less than five days) after insertion, but only 46% of them sought treatment within a month. The majority were symptomatic on presentation (71%), with most symptoms being either abnormal bleeding, mild lower abdominal pain, or both [8]. As symptoms are non-specific and mostly mild, they could go undiagnosed or be overlooked easily. Thus, clinicians need to be made aware of such complications and perform careful examinations of patients with missing IUCD threads and non-specific symptoms.

Here, we present the case of an unusual presentation of a migrated copper IUCD into the rectosigmoid junction.

## Case presentation

**Figure 1:**
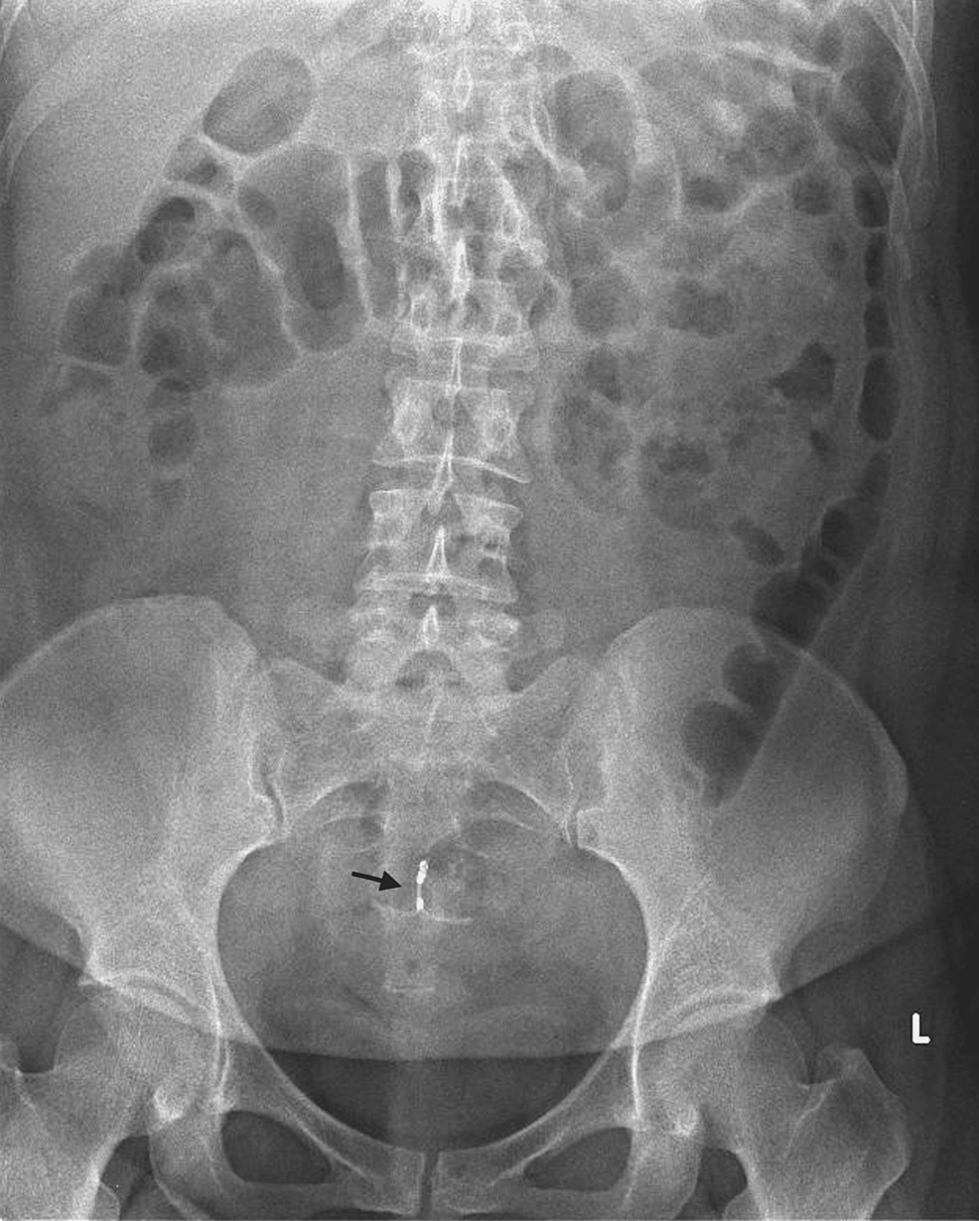
An X-ray of the KUB shows an intrauterine device (arrow) noted within the midline of the pelvis. No renal stones were noted. KUB: kidney, ureter, and bladder

A 33-year-old woman (parity one) presented to the emergency department with a three-day history of right loin-to-groin pain associated with a low-grade temperature of 37.6 degrees Celsius and dysuria. She denied any bowel-related symptoms. She had presented to her general practitioner with urinary symptoms two days prior and was started on oral antibiotics for a presumed lower urinary tract infection. As her symptoms persisted and compounded with right loin-to-groin pain and fever, she presented to the emergency department. On examination in the emergency department, she had tenderness over the right hypochondrium and right flank with no signs of peritonitis. An X-ray of the kidney, ureter, and bladder done in the emergency department showed no renal stones, and an intrauterine device was noted within the midline of the pelvis (Figure 1).

Her investigations demonstrated a raised total white cell count of 11.3 x 109/L, and she was subsequently commenced on intravenous antibiotics and analgesia.

**Figure 2:**
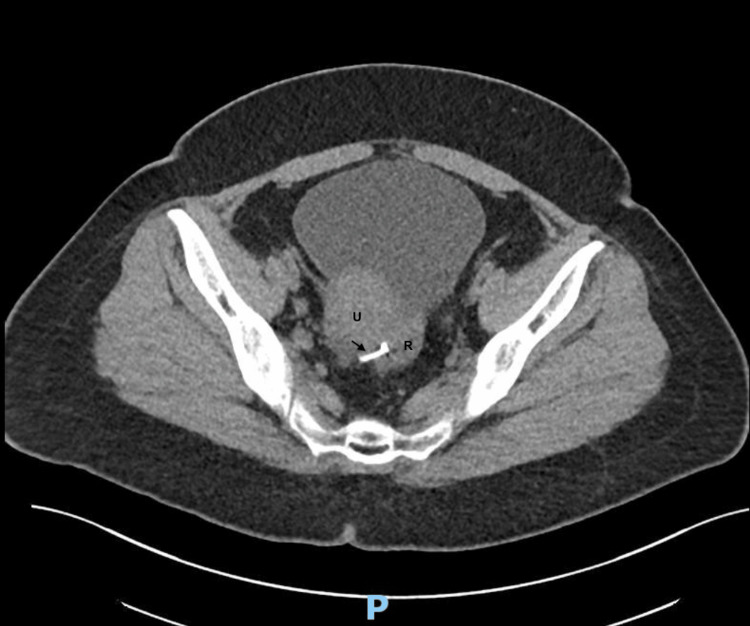
A CT scan of the KUB (transverse view) shows the intrauterine device (arrowed) has migrated and is now located between the body of the uterus (labelled U) and the adjacent rectosigmoid colon (labelled R). KUB: kidney, ureter, and bladder

**Figure 3:**
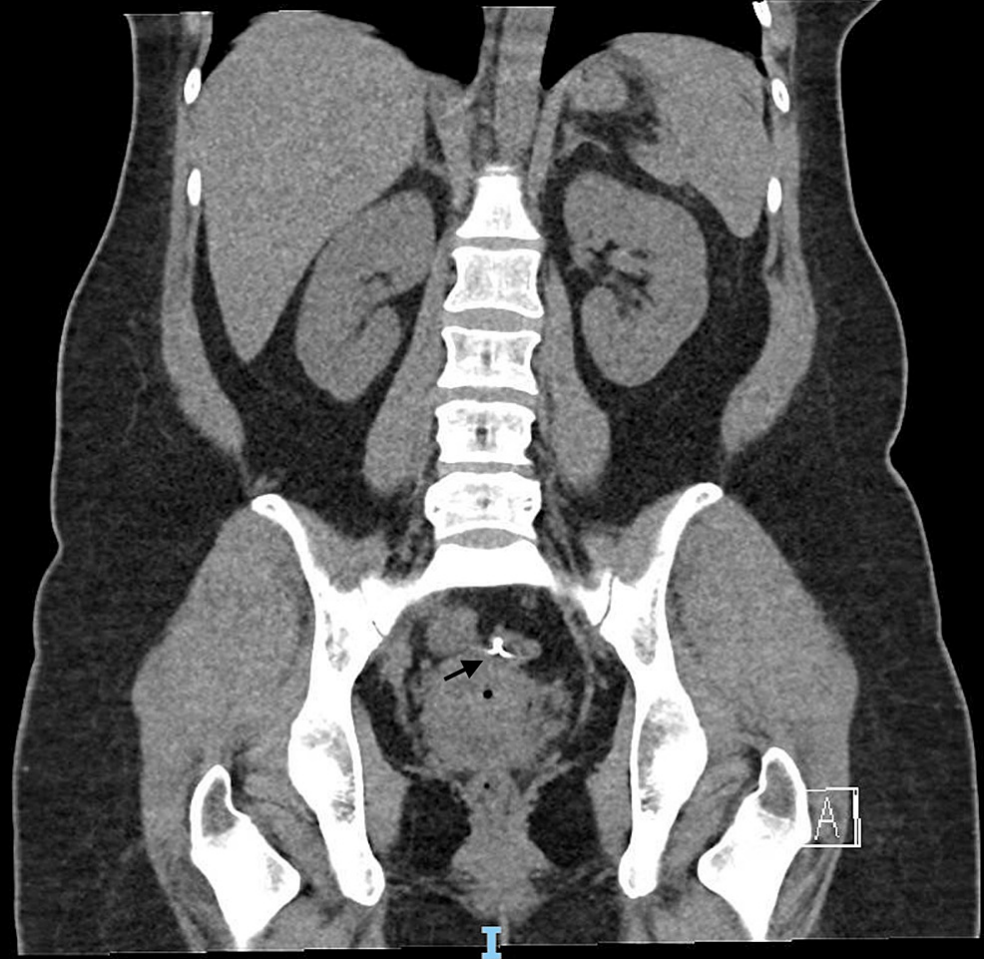
A CT scan of the KUB (coronal view) shows the intrauterine device (arrowed) has migrated and is now located between the body of the uterus and the adjacent rectosigmoid colon. KUB: kidney, ureter, and bladder

Despite treatment, her symptoms persisted, and thus further imaging tests were performed. A subsequent computed tomography of the kidneys, ureters, and bladder (CT-KUB) performed reported a migrated intrauterine device located between the body of the uterus and the adjacent rectosigmoid colon. There were small pockets of gas surrounding it with no fluid collection, and no calculus was seen in the urinary tract. (Figure 2-3).

Magnetic resonance imaging of the pelvis (MRI-pelvis) subsequently confirmed a migrated IUCD posterior to the uterine body, with the horizontal arms of the IUCD closely abutting the adjacent proximal rectum.

On further history, the patient reported having had a copper intrauterine device inserted five years ago postnatally after an emergency caesarean section for failure to progress. During her fourth-week postnatal visit, she had a Papanicolaou (PAP) smear done, which showed normal endocervical cells with no malignancy, and her copper IUCD was inserted in the same setting. The patient was planned to be followed up on in two months. However, she defaulted on her clinic appointment and was unable to follow up thereafter. Two years later, the patient was residing overseas and presented to the doctors there with a complaint of amenorrhea. During the consultations, she was informed of the migrated IUCD and underwent diagnostic laparoscopy. After the diagnostic laparoscopy, she was told by the surgeons that, due to the location of the IUCD, there was a high risk of bowel perforation if removal was attempted. As it was a small district hospital with no other specialty support, the decision was made to abandon the procedure and leave the IUCD in. She was advised to seek medical attention if she had any abdominal pain and remained well until her presentation to our institution.

**Figure 4:**
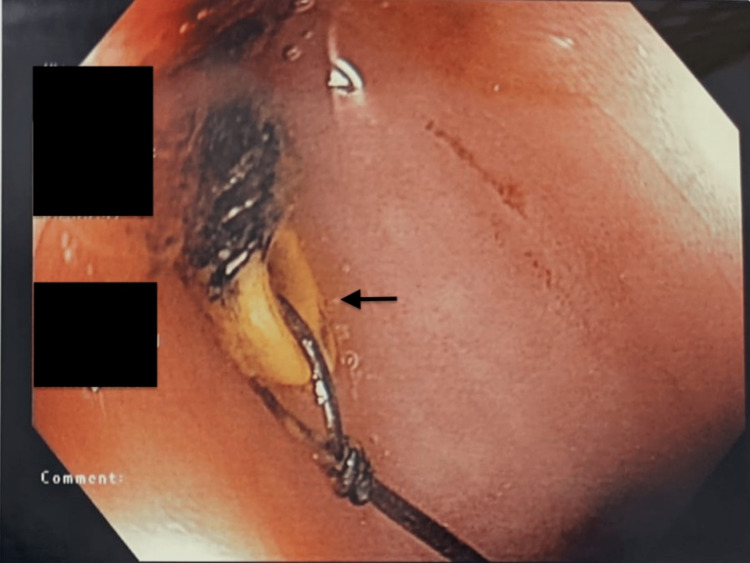
Flexible sigmoidoscopy view of the migrated IUCD (arrow) IUCD: intrauterine contraceptive device

When she was referred to the gynaecologists, due to the involvement of the bowels, the management of the patient was done in collaboration with the colorectal surgeons. In view of the persistent abdominal pain and known findings of a migrated IUCD, the patient opted for surgical management. Preoperatively, she underwent a flexible sigmoidoscopy, which demonstrated an erosion of the distal tip of the IUCD into the rectosigmoid junction. The IUCD was intact with the wire present, approximately 18 cm from the anal verge on the patient’s right anterolateral region, as shown in Figure 4.

She was then prepared for surgery for the removal of the IUCD.

**Figure 5:**
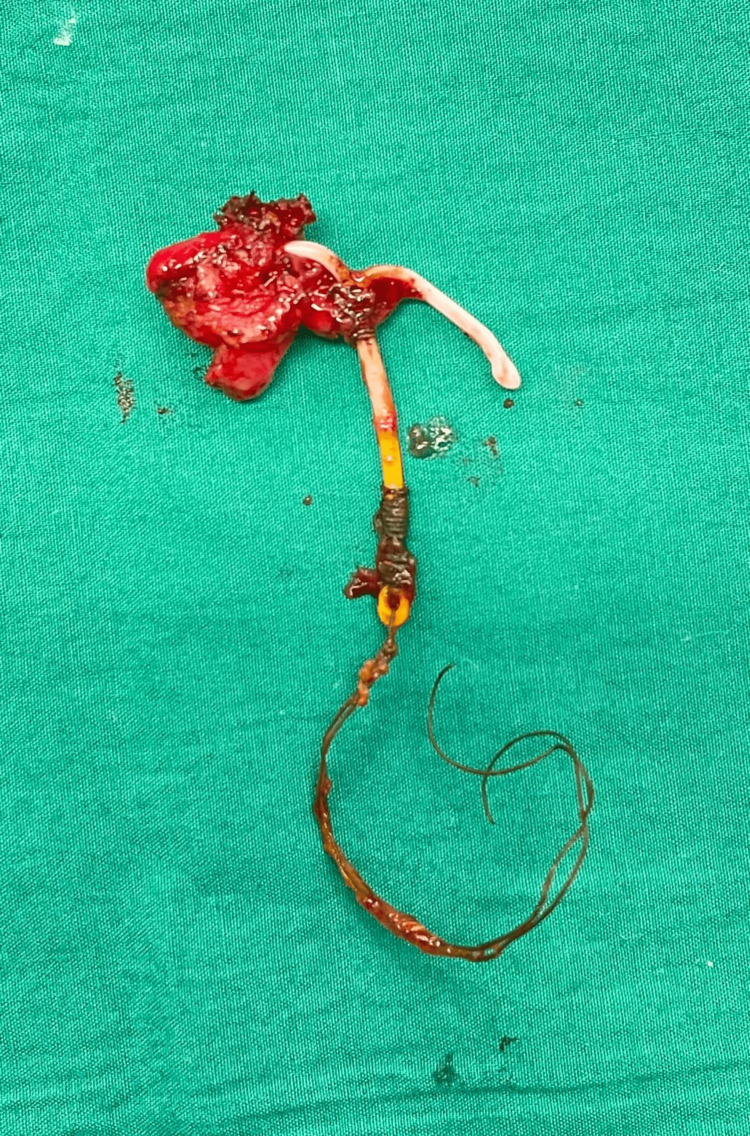
Removed IUCD post partial rectotomy. The “T” of IUCD was noted to be embedded external to the recto-sigmoid junction with surrounding chronic inflammation. IUCD: intrauterine contraceptive device

Intraoperatively, her hysteroscopy noted a normal-sized uterus with an intact cavity with no IUCD seen inside. She underwent another flexible sigmoidoscopy, which demonstrated an erosion of the distal tip of the IUCD into the rectosigmoid junction. Gentle traction to attempt removal of the IUCD failed, and hence a diagnostic laparoscopy was performed to remove the impacted IUCD intra-abdominally with the repair of the defect in the rectum. Laparoscopically, the horizontal arms of the IUCD were noted to be embedded externally in the recto-sigmoid junction with surrounding chronic inflammation. The uterus was otherwise completely clear of the rectum, with no fistula noted. A partial rectotomy was done to remove the embedded IUCD, and the defect was closed in two layers with Vicryl 3-0 sutures. The migrated intrauterine device was eventually removed completely, as seen in Figure 5.

The patient recovered well postoperatively and was discharged two days later. On her first follow-up review in the outpatient clinic after discharge, the patient remained well with no abdominal pain. She continues to be followed up in both the colorectal and gynaecology clinics.

## Discussion

The intrauterine contraceptive device (IUCD) is one of the most effective and reliable contraceptives used worldwide. The migration of IUCDs is rare. Most cases present with mild symptoms of either abnormal bleeding or mild lower abdominal pain, or both [8]. Other cases were asymptomatic patients diagnosed during routine follow-up visits (migrated IUCD suspected due to missing threads on speculum examination) or because of an unintended pregnancy. One intra-abdominal IUCD was found during a hysterectomy performed for other reasons, and the patient had forgotten the presence of the IUCD [8]. As symptoms are non-specific and mostly mild, they could go undiagnosed easily. In our case report, our patient remained asymptomatic for five years prior to undergoing a definitive operation to remove the migrated IUCD. She eventually presented unusually with right-sided pain and urinary symptoms and underwent a few imaging procedures prior to her eventual diagnosis. As such, clinicians need to be made aware of complications from IUCD insertion and perform careful examinations of patients with missing IUCD threads and non-specific symptoms.

A review of the literature noted that the risk of uterine perforation is increased in a retroverted uterus if insertion is done during the early postpartum state or if the patient was breastfeeding at the time of insertion [2, 4, 6]. Andersson et al. suggested that oestrogen levels are low during lactation, which results in a small uterus, thus increasing the risk of uterine perforation [2]. An increased pain threshold from increased beta-endorphins in lactating women has also been suggested as the cause of unnoticed uterine perforation during IUCD insertion in a lactating woman [9]. Some literature suggests postponing IUCD insertion to six months postpartum rather than three months due to the risk of pregnancy, but this will require further study prior to routine recommendations [9-11]. 

Migration or translocation of the IUCD into adjacent organs can have serious consequences. Translocation of the IUCD into the bladder, caecum, sigmoid, appendix, and small bowel have all been described in the literature [7, 12-16]. There have also been reports of colocolonic fistulas, which occur when one limb of the IUCD is in the sigmoid while the other limb perforates an adjacent loop of the transverse colon [17]. Reports of volvulus, bowel obstruction, and death from complications of IUCD placement have also been noted in the literature [18]. When an IUCD is noted to have migrated, an exploratory laparoscopy (if suitable) or laparotomy is often the first line of intervention. This is typically recommended due to the potential sequalae of a migrated IUCD. However, there are some authors who have suggested that non-operative management could be an option for asymptomatic patients because of the morbidity associated with surgery and anaesthesia [19]. This remains debatable, and further research needs to be done before formal recommendations can be proposed.

In addition, the method of IUCD retrieval depends on the location of the migrated IUCD. There are reports of alternative retrieval methods for partially embedded IUCD in the sigmoid colon or rectum, which may provide a less invasive method if appropriate and thus avoid the need for exploratory laparoscopy or laparotomy. In a review by Assarian and Raja, they reported four cases in the literature whereby the migrated IUCD in the rectosigmoid region were removed by manual traction, five cases with the help of a proctoscope, three other cases with the help of rigid sigmoidoscopy, and one case where a colonoscope was used and the migrated device was removed by a snare [20]. They suggested the consideration of colonoscopic or transrectal retrieval as potential feasible methods for retrieval and routine preoperative endoscopic evaluation to be done for similar cases to avoid unnecessary invasive surgical intervention [20]. Our patient underwent a preoperative endoscopic evaluation of the migrated IUCD. However, in conjunction with the imaging and endoscopic findings, the IUCD was deemed to be too deeply impacted, and thus no endoscopic retrieval was attempted preoperatively. Subsequently, she underwent surgery, and an endoscopic retrieval was attempted intraoperatively under general anaesthesia. This, however, was unsuccessful. Our patient eventually had a diagnostic laparoscopy with a partial rectotomy to remove the embedded IUCD, and the defect was closed in two layers with Vicryl 3-0 sutures. As such, endoscopic retrieval should be attempted only on a case-by-case basis.

Due to the involvement of multiple organs, it is prudent to involve other specialists for a multidisciplinary approach in the care of such cases. Management should be based on a combination of radiological findings, endoscopic findings, and knowledge of the anatomy of the abdominal organs. In our case, the management was heavily reliant on the coordination between the colorectal surgeons and gynaecologists. We also employed a multifaceted approach, with the assistance of radiological imaging and endoscopic images preoperatively, for preoperative planning and counselling for this patient.

## Conclusions

This case presents many points for discussion regarding the management of a migrated IUCD. Many innovative ways were discussed regarding its assessment, which includes preoperative imaging or endoscopic evaluation. Novel methods of removal of migrated IUCD in the rectosigmoid colon, for example, with the use of a proctoscope or colonoscope, have been proposed as alternatives to the traditional diagnostic laparoscopy or laparotomy, reducing the need for general anaesthesia or operative intervention. Looking forward, long-term studies can be done to evaluate the need for intervention for asymptomatic patients where the risk of surgery may outweigh the benefits. Non-invasive surgical approaches could also be explored as alternatives to the management of a migrated IUCD. Albeit a rare complication from IUCD insertion, this case highlights the need for clinicians to be aware of potential complications arising from IUCD insertion and its subsequent management. It also emphasises the need for thorough counselling of patients regarding symptoms of abdominal pain and abnormal menses post-IUCD insertion and reiteration of compliance to follow-up after IUCD insertion.
